# Acute Exposure to Crystalline Silica Reduces Macrophage Activation in Response to Bacterial Lipoproteins

**DOI:** 10.3389/fimmu.2016.00049

**Published:** 2016-02-15

**Authors:** Gillian L. Beamer, Benjamin P. Seaver, Forrest Jessop, David M. Shepherd, Celine A. Beamer

**Affiliations:** ^1^Department of Infectious Diseases and Global Health, Cummings School of Veterinary Medicine, Tufts University, North Grafton, MA, USA; ^2^Department of Biomedical and Pharmaceutical Sciences, University of Montana, Missoula, MT, USA; ^3^Center for Environmental Health Sciences, Missoula, MT, USA; ^4^Center for Biomolecular Structure and Dynamics, Missoula, MT, USA

**Keywords:** lung, inflammation, mouse, scavenger receptor, toll-like receptor, bacterial susceptibility

## Abstract

Numerous studies have examined the relationship between alveolar macrophages (AMs) and crystalline silica (SiO_2_) using *in vitro* and *in vivo* immunotoxicity models; however, exactly how exposure to SiO_2_ alters the functionality of AM and the potential consequences for immunity to respiratory pathogens remains largely unknown. Because recognition and clearance of inhaled particulates and microbes are largely mediated by pattern recognition receptors (PRRs) on the surface of AM, we hypothesized that exposure to SiO_2_ limits the ability of AM to respond to bacterial challenge by altering PRR expression. Alveolar and bone marrow-derived macrophages downregulate TLR2 expression following acute SiO_2_ exposure (e.g., 4 h). Interestingly, these responses were dependent on interactions between SiO_2_ and the class A scavenger receptor CD204, but not MARCO. Furthermore, SiO_2_ exposure decreased uptake of fluorescently labeled Pam_2_CSK_4_ and Pam_3_CSK4, resulting in reduced secretion of IL-1β, but not IL-6. Collectively, our data suggest that SiO_2_ exposure alters AM phenotype, which in turn affects their ability to uptake and respond to bacterial lipoproteins.

## Introduction

Silicon dioxide, also known as silica, is one of the most common elements on earth, yet its inhalation can result in acute lung injury and ongoing inhalation can result in permanent lung damage due to deposition of particles in the lung. Silicosis is a progressive, disabling, and often-fatal lung disease resulting from the inhalation of crystalline silica (SiO_2_) particles over prolonged periods of time. Silicosis occurs as the result of exposure through occupation (e.g., construction, mining), recreation (e.g., pottery), or environment (e.g., soil). Inhalation of SiO_2_ particles causes a granulomatous inflammatory response that progresses to interstitial fibrosis as well as systemic immune deficits (1–5). There is no cure for silicosis, and treatment options are limited. Although significant efforts have been made through industrial hygiene standards to control ambient dust in the workplace, silicosis remains a prevalent health problem throughout the world, particularly in developing nations (6).

In addition to its importance as an occupational hazard, inhalation of SiO_2_ predisposes workers to bacterial infections, impairs lung defense mechanisms, and significantly shortens worker lifespans – particularly in less-advanced countries and among disadvantaged persons in developed nations (1, 7). In particular, SiO_2_-exposed workers, with or without silicosis, are at increased risk for tuberculosis and non-tuberculous mycobacteria-related diseases (5, 8, 9). Previous studies suggest that the acute and accelerated forms of silicosis exhibit the highest prevalence of silicotuberculosis (1), and that the development of *Mycobacterium tuberculosis* (Mtb) infection is directly dependent on the collective SiO_2_ exposure (5, 8, 10). Indeed, SiO_2_ exposure results in a threefold or greater risk of developing pulmonary Mtb infections. Similarly, recent studies demonstrate that acute exposure to silica nanoparticles increases the susceptibility of mice to *Pseudomonas aeruginosa*-induced pneumonia (11).

Alveolar macrophages (AMs) play a critical role in the ongoing cross-talk between innate and adaptive immune responses in the lung and are the typical host cell for an array of pathogens such as bacterial infections (e.g., Mtb) and many airborne particulates (e.g., SiO_2_). In macrophages, formation and activation of the NLRP3 inflammasome are an important mechanism mediating the inflammatory response to numerous particulates, including nanoparticles, silica, MSU crystals, asbestos, and urban particulate matter, resulting in promotion of IL-1β release (12–15). Macrophages not only initiate the inflammatory process to SiO_2_ (16) but also play an important role in host resistance to bacterial infections, including Mtb (17). Moreover, numerous adverse effects on macrophage function have been described following exposure to SiO_2_ (18–27), suggesting that SiO_2_-mediated macrophage injury might impair host defense and increase susceptibility to infection. The current view is that SiO_2_ “damages” macrophages or alters their phenotype, thereby inhibiting their ability to phagocytose and kill bacteria (19, 28); however, the molecular mechanisms underlying this predisposition remain unknown.

Alveolar macrophages sense bacterial pathogens through pattern recognition receptors (PRRs) (29, 30) *via* the detection of highly conserved molecular structures, designated pathogen-associated molecular patterns (PAMPs) (31–33). Numerous families of PRRs exist, all of which recognize a different repertoire of PAMPs, including C-type lectin receptors, scavenger receptors (SRs), toll-like receptors (TLRs), NOD-like receptors, and RIG-I-like receptors. Of these PRRs, TLRs 1/2, 4, and C-type lectin receptors have all been shown to mediate the *in vitro* recognition of Mtb and the cytokine response of macrophages is lower in the absence of these TLRs (34). It is clear that macrophages contribute to the lung response to SiO_2_ and to bacterial infections independently, and that these effects may be mediated through PRRs. Therefore, we investigated whether SiO_2_ exposure alters the expression of select PRRs on alveolar and bone marrow-derived macrophages and assessed the ability of SiO_2_-exposed macrophages to uptake and respond to bacterial lipoproteins acting at TLR2/1 and TLR2/6.

## Materials and Methods

### Mice

Breeding pairs of C57BL/6 (C57BL/6J, stock #000664) mice were originally purchased from The Jackson Laboratory (Bar Harbor, ME, USA); whereas breeding pairs of MARCO^−/−^ and SRA^−/−^ mice on C57BL/6 background were kindly provided by Dr. Lester Kobzik (Harvard School of Public Health, Boston, MA, USA), and caspase 1-deficient (casp1^−/−^ B6N.129S2-Casp1^tm1Flv^/J, stock #016621) mice (for experimental use) were kindly provided by Dr. Andrij Holian (University of Montana, Center for Environmental Health Sciences). All mice were maintained in the University of Montana Specific Pathogen-Free (SPF) Laboratory Animal Facility and both sexes used at 6–8 weeks of age. All animal use procedures were in accordance with NIH and University of Montana IACUC guidelines.

### Experimental Instillations

Crystalline silica (SiO_2_, 1.5–2 μm) (Pennsylvania Glass Sand Corporation, Pittsburgh, PA, USA) was acid washed, dried, and determined to be free of endotoxin (data not shown). Mice were anesthetized with isoflurane and instilled *via* the intranasal (i.n.) exposure route with 25 μl sterile saline (vehicle) or 1 mg SiO_2_ suspended in 25 μl of sterile saline (35, 36). Mice were then returned to their cages and monitored until mobility returned. Whole lung lavage samples were collected at 4, 24, and 72 h, as well as 7 days following the initial instillation, as previously described (37, 38).

### Flow Cytometry

Single cell suspensions from either whole lung lavages or bone marrow-derived macrophages were washed and re-suspended in 100 μl of purified rat anti-mouse CD16/CD32 diluted 1:100 in PBS with 1% bovine serum albumin and 0.1% sodium azide (PAB) for 15 min on ice to block non-specific Ab binding. Monoclonal Abs specific to CD11c redFluor 710 (clone #N418, Tonbo Biosciences), F4-80 FITC (clone BM-8), DC-SIGN PE (clone # 5H10), TLR2 eF450 (clone # 6C2 eBiosciences), TLR4 PE-Cy7 (clone # SA15-21, Biolegend), TLR5 AF647 (clone # ACT5), and TLR6 (clone # 418601, R&D Systems) to identify cell surface receptor density on live, F4-80^+^CD11c^+^ AMs (23). Following titration of the individual antibodies in preliminary experiments, 1 μg of each Ab was added per 10^6^ total cells and allowed to incubate for 30 min in the dark on ice, with agitation two to three times. Finally, cells were washed twice with PBS and re-suspended in 0.3 ml PAB on ice. Immediately before acquisition, 5 μl of propidium iodide solution (BioLegend) was added per 10^6^ total cells and allowed to incubate for 15 min prior to analysis. Cell acquisition and analysis were performed on a FACS Aria flow cytometer using FACS Diva software (version 6.1.2, Becton Dickinson), with the exception of Figure 4 – where cell acquisition and analysis was performed on a Attune NxT Accoustic Focusing Cytometer using Attune NxT software (version 2.2, Thermo Fisher Scientific). In the multi-color staining panels, positive/negative gates were set based on fluorescence minus one (FMO) controls and checked against single stained controls. Compensation of the spectral overlap for each fluorochrome was performed using compensation control beads (BD Biosciences).

### Generation and Stimulation of Bone Marrow-Derived Macrophages

Bone marrow macrophages (BMM) were generated using murine recombinant macrophage colony-stimulating factor (50 ng/ml, U.S. Biological, Swampscott, MA, USA), as previously described (37, 39). By 7 days, cells were fully differentiated, >75% confluent, and immune-positive for macrophage characteristics (F4-80^+^CD11b^+^ MHC class II^low^), as assessed by flow cyometry (data not shown). Viability was determined to be >90% by trypan blue exclusion staining prior to experimental manipulations. BMM were seeded at 10^6^ cells/ml/well of a 6-well plate, immediately exposed to media alone (vehicle) or 50–100 μg/ml SiO_2_, and allowed to incubate for 4 or 24 h at 37°C. By 24 h at these exposure levels, ~20% of the BMM exhibited signs of apoptosis and/or cell death using trypan blue exclusion and/or live dead dyes during flow cytometry experiments. Following stimulation, BMM were lightly scraped within the spent culture media, centrifuged, and the supernatant and cells separated for analysis.

### Confocal Microscopy and Quantification of Uptake of Rhodamine TLR Ligands

Bone marrow macrophages (1 × 10^6^ cells/eppendorf microfuge tube) were exposed to media alone (vehicle) or 50–100 μg/ml SiO_2_ on a rotisserie for 24 h at 37°C. Macrophages were subsequently exposed to 0.5 μg/ml rhodamine conjugated Pam_2_CSK_4_ (TLR2/6 ligand) or Pam_3_CSK4 (TLR2/1 ligand) (Invivogen) for 2 h. Cells were washed twice with PBS and were cytospun (1 × 10^5^) onto glass slides, coverslipped with Prolong Gold with Dapi, and images collected on an Olympus Fluoview Confocal Imaging System. NIH Image J software or flow cytometry was used to analyze mean fluorescence intensity (MFI) and side scatter (SSC) properties of the BMMs to measure TLR ligand or SiO_2_ uptake (40).

### Cytokine ELISAs

IL-1β, TNFα, IL-6, and IL-10 were measured in tissue culture supernatants using murine ELISA kits according to the manufacturer’s instructions and assay procedure (R&D Systems). Color development was assessed at 450 nm on a plate reader.

### Statistical Analysis

For each parameter, the values for individual mice were averaged and the SD and SE calculated. The significance of the differences between the exposure groups was determined by *t*-test, one-way, or two-way ANOVA, in conjunction with Tukey’s test for variance, where appropriate. All ANOVA models were performed with Prism software, version 4. A *p*-value of <0.05 was considered significant.

## Results

### Differential Pattern Recognition Receptor Expression following Acute Silica Exposure

**Figure 1:**
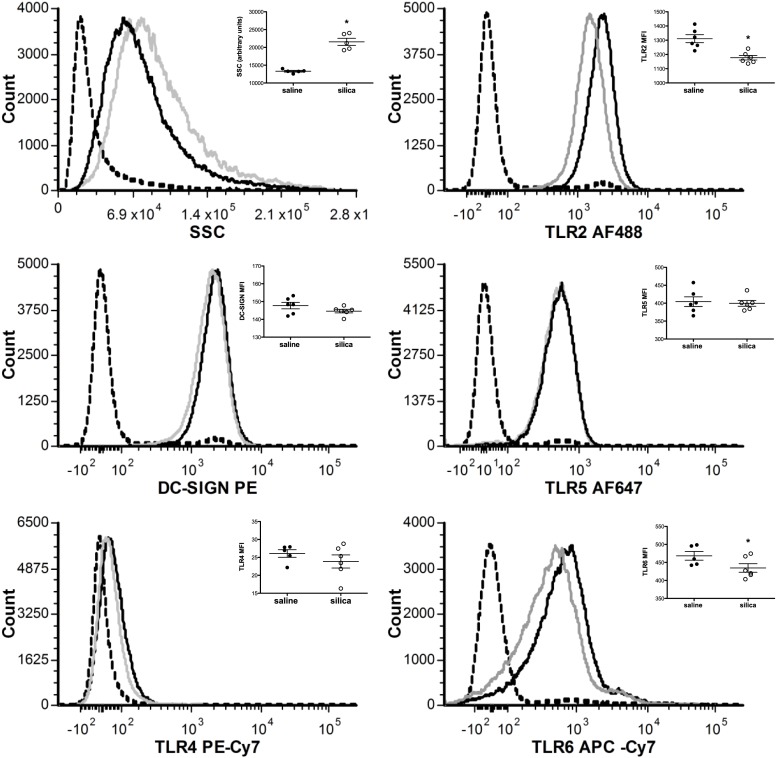
**Acute silica exposure selectively reduced TLR2 and TLR6 expression on F4-80^+^CD11c^+^ alveolar macrophages**. C57Bl/6 wild-type mice were exposed to saline (25 μl, black line) or silica (1 mg, gray line) through intranasal aspiration. After 4 h, whole lungs were lavaged and cells immunostained. Representative histograms from the flow cytometric analysis demonstrates concomitant uptake of SiO_2_ particles through increases in side scatter (SSC) measurements and decreased expression of TLR2 and TLR6 expression on alveolar macrophages (AMs) relative to saline control. By contrast, no change was observed in the cell surface expression of DC-SIGN, TLR4, or TLR5 on live, F4-80^+^CD11c^+^AMs in response to SiO_2_. Scatter plots (inset) show the raw data in graphical form. Results are means ± SEM (*n* = 6). *p < 0.05 compared to saline.

Previous studies established that SiO_2_ alters the phenotype and function of AM, bone marrow-derived dendritic cells, and macrophages, and freshly isolated interstitial macrophages and dendritic cells (23, 39, 41); however, these studies did not evaluate PRR expression on AM in response to SiO_2_. To test whether exposure of AMs *in situ* resulted in altered expression of PRRs, C57Bl/6 wild-type mice were instilled with either saline (vehicle control) or 1 mg SiO_2_. Four hours after encountering SiO_2_ in the alveolus, flow cytometry confirmed that live (PI negative) F4-80^+^CD11c^+^AMs had taken up SiO_2_ particles *via* changes in SSC properties, and simultaneously downregulated their expression of TLR2 and TLR6, but not DC-SIGN, TLR4, or TLR5 (Figure 1, inset). Representative histograms illustrate the relative change in fluorescent intensity between AMs lavaged from the airways of saline (black line) vs. SiO_2_ (silver line) exposed C57Bl/6 wild-type mice, compared to unstained controls (dashed line) (Figure 1).

**Figure 2:**
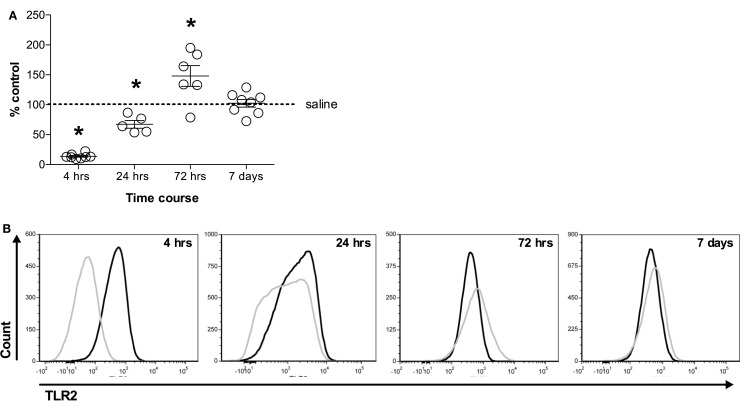
**Silica exposure altered TLR2 expression on F4-80^+^CD11c^+^ alveolar macrophages**. C57Bl/6 wild-type mice were exposed to saline (25 μl) or silica (1 mg) through intranasal aspiration. After 4, 24, 72 h, and 7 days, whole lungs were lavaged and cells immunostained. **(A)** Flow cytometric analysis demonstrates decreased expression of TLR2 on live, F4-80^+^CD11c^+^ alveolar macrophages (AMs) at 4 and 24 h, whereas TLR2 expression was increased on AMs at 72 h. By contrast, no change was observed in the cell surface expression of TLR2 on AMs 7 days following exposure to SiO_2_. Results are presented as mean percent of control ± SEM (*n* = 6-8). *p < 0.05 compared to saline control. **(B)** Representative histograms from the flow cytometric analysis demonstrate changes in the cell surface expression of TLR2 as a function of time.

In the murine model, TLR-2 in particular plays a crucial role in the cellular response to bacterial pathogens. Therefore, we evaluated whether SiO_2_ exposure downregulated TLR2 expression levels on live, F4-80^+^CD11c^+^ AMs in a time-dependent manner in wild-type C57BL/6 mice. SiO_2_ exposure reduced TLR2 expression on the surface of AMs by 86.4 and 32.9% relative to their corresponding saline controls at 4 and 24 h, respectively. By contrast, SiO_2_ exposure increased TLR2 expression on the surface of AMs by 47.8% relative to the saline control at 72 h. By 7 days after silica exposure, TLR2 expression on live, F4-80^+^CD11c^+^ AMs had returned to baseline (Figure 2A). Representative histograms illustrate the relative change in fluorescent intensity between saline (black line) and SiO_2_ (silver line) exposed mice at the indicated time point following exposure (Figure 2B).

### Silica-Induced Changes in TLR2 Expression Are Dependent on CD204, but Not Inflammasome Activation

**Figure 3:**
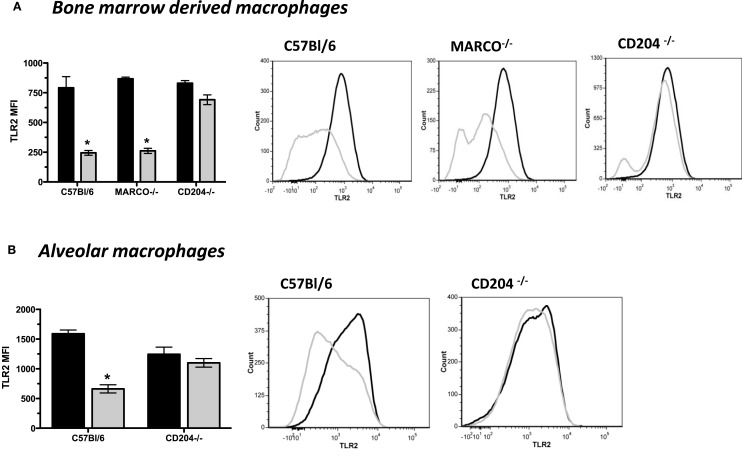
**Silica-induced decrease in TLR2 expression is dependent on the scavenger receptor CD204 *in vitro* and *in vivo***. **(A)** Macrophages were derived from the bone marrow of wild-type C57Bl/6, CD204^−/−^, and MACRO^−/−^ mice and subsequently exposed to media or silica (100 μg/ml). As anticipated, flow cytometric analysis demonstrates decreased expression of TLR2 on *live*, F4-80^+^CD11b^+^ macrophages 4 h following exposure to SiO_2_. This reduction in the cell surface expression of TLR2 was dependent on the presence of the scavenger receptor CD204, but not MARCO. Results are means ± SEM (*n* = 3–5). *p < 0.05 compared to saline. Representative histograms from the flow cytometric analysis display changes in the cell surface expression of TLR2 as a function of mouse strain. **(B)** Wild-type C57Bl/6 and CD204^−/−^ mice were exposed to saline (25 μl) or silica (1 mg) through intranasal aspiration. After 4 h, whole lungs were lavaged and cells immunostained and analyzed by flow cytometry. Flow cytometry shows decreased expression of TLR2 on *live*, F4-80^+^CD11bc^+^ AMs 4 h after SiO_2_, which is dependent on the presence of the scavenger receptor CD204. Results are means ± SEM (*n* = 4–5). *p < 0.05 compared to saline. Representative histograms from the flow cytometric analysis display changes in the cell surface expression of TLR2.

Previous studies from our laboratory established that the class A SRs CD204 and MARCO were important for the binding/uptake of SiO_2_ and subsequent inflammatory response (35, 40). Therefore, we investigated the relationship between CD204 and MARCO, and SiO_2_-induced changes in TLR2 expression. Using bone marrow-derived macrophages as a model system (41), we demonstrate that SiO_2_-induced loss of TLR2 expression on F4-80^+^CD11b^+^ macrophages was dependent on CD204, but not MARCO at 4 h (Figure 3A) and 24 h post-exposure (data not shown). Representative histograms illustrate the relative change in fluorescent intensity of TLR2 on the cell surface of media (black line) and SiO_2_ (silver line)-treated bone marrow-derived macrophages (Figure 3A). These results were confirmed using AMs lavaged from saline and SiO_2_-exposed C57Bl/6 wild-type and CD204^−/−^ mice, at 4 h post-exposure (Figure 3B). Representative histograms illustrate the relative change in fluorescent intensity of TLR2 on the cell surface of live, F4-80^+^CD11c^+^ AMs between saline (black line) and SiO_2_ (silver line) exposed mice (Figure 3B).

**Figure 4:**
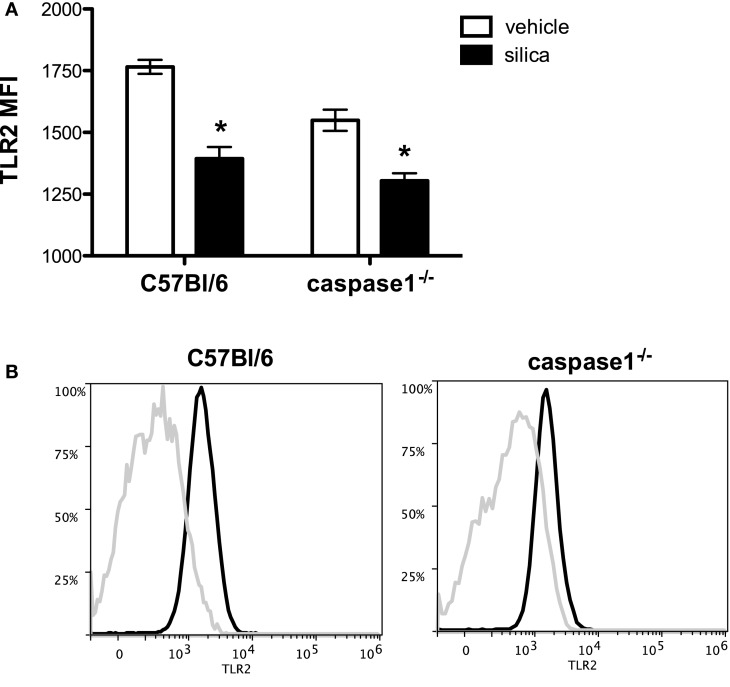
**Silica-induced reduction in TLR2 expression occurs independently of inflammasome activation**. Wild-type C57Bl/6 and caspase 1^−/−^ mice were exposed to saline (25 μl) or silica (1 mg) *via* intranasal aspiration. After 4 h, whole lungs were lavaged and cells immunostained and analyzed by flow cytometry using the Attune NxT acoustic focusing flow cytometer. **(A)** Flow cytometry corroborates decreased expression of TLR2 on *live*, F4-80^+^CD11c^+^ AMs isolated from C57Bl/6 mice 4 h after SiO_2_ and further demonstrates that this decrease is not dependent on the NLRP3 inflammasome and IL-1β secretion. Results are means ± SEM (*n* = 3–4, repeated twice). *p < 0.05 compared to saline. **(B)** Representative histograms from the flow cytometric analysis demonstrate changes in the cell surface expression of TLR2 as a function of time.

NLRP3 inflammasome activation and resultant IL-1β production by AMs is recognized as a significant mechanism underlying silicosis (13, 24, 26, 42). Because activation of the NLRP3 inflammasome converges on caspase 1, which then contributes to the production and secretion of mature IL-1β, we examined the contributions of inflammasome activation to SiO_2_-induced changes in TLR2 expression using caspase 1-deficient (caspase 1^−/−^) mice. Four hours following exposure, we show that SiO_2_-induced loss of TLR2 expression on live, F4-80^+^CD11c^+^ AMs occurs independent from NLRP3 inflammasome activation and secretion of IL-1β (Figure 4A). Representative histograms demonstrate the relative change in fluorescent intensity of TLR2 on the cell surface of live, F4-80^+^CD11c^+^ AMs between saline (black line) and SiO_2_ (silver line) exposed C57Bl/6 and caspase 1^−/−^ mice (Figure 4B).

### Effects of Silica Exposure on the Uptake of TLR2/1 and TLR2/6 Ligands

**Figure 5:**
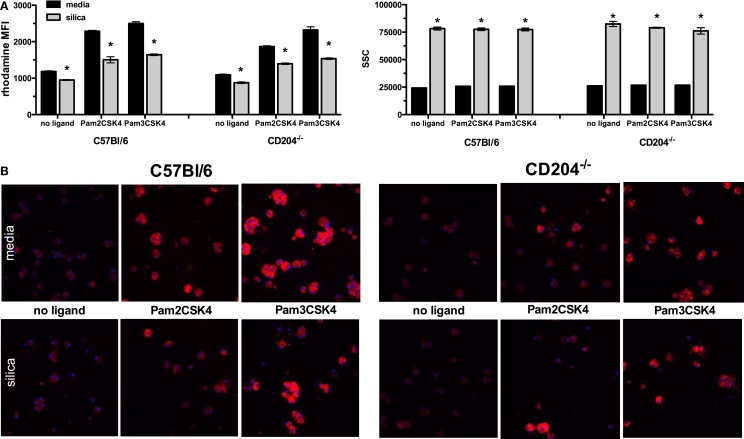
**Silica exposure decreased uptake of fluorescently labeled synthetic diacylated and triacylated lipoproteins *in vitro***. Macrophages derived from the bone marrow of wild-type C57Bl/6 and CD204^−/−^ mice were exposed to media or silica (100 μg/ml) for 24 h and subsequently treated with fluorescently labeled Pam_2_CSK_4_ and Pam_3_CSK4 for 2 h. **(A)** Flow cytometry revealed uptake of the rhomadine-labeled lipoproteins into macrophages as measured by an increase in the mean fluorescence intensity (MFI), which was attenuated in the SiO_2_-exposed macrophages. This increase in MFI was not due to changes in the uptake of SiO_2_ into the macrophages, as shown by an increase in SSC. **(B)** Representative images from confocal microscopy showed increased fluorescence in the macrophages exposed to fluorescently labeled lipoproteins vs. media alone. Moreover, this fluorescence was diminished in SiO_2_-exposed macrophages. Results are means ± SEM (*n* = 5). *p < 0.05 compared to media.

Because TLR2 cooperates with TLR6 in response to diacylated mycoplasmal lipopeptide and associates with TLR1 to recognize triacylated lipopetides, we next examined the ability of SiO_2_-exposed bone marrow-derived macrophages to take up fluorescently labeled bacterial cell wall components recognized by the TLR2/1 heterodimer (Pam_3_CSK_4_) and TLR2/6 heterodimer (Pam_2_CSK_4_) using a combination of flow cytometry and confocal microscopy. Flow cytometry demonstrated that SiO_2_ exposure reduced the uptake (e.g., MFI) of both the synthetic triacylated and synthetic diacylated lipoproteins recognized by TLR2/1 and TLR2/6 heterodimers, respectively, in both C57Bl/6 and CD204^−/−^ derived cells (Figure 5A). This response was slightly dampened in CD204^−/−^ cells vs. C57Bl/6 cells. Moreover, simultaneous measurements of SSC characteristics revealed comparable levels of SiO_2_ uptake across all exposure groups and mouse strains (Figure 5A), indicating similar levels of SiO_2_ exposure and uptake across treatment groups. Representative images collected *via* confocal microscopy support the observation of reduced uptake of fluorescently labeled TLR ligands in response to SiO_2_ exposure (Figure 5B). These changes were quantified by image analysis using NIH Image J and showed a similar reduction in uptake of fluorescently labeled diacylated and triacylated lipopetides into SiO_2_-exposed cells (data not shown).

### Silica Exposure Reduced IL-1β Levels in Response to Synthetic Triacylated and Synthetic Diacylated Lipoproteins *In Vitro*

**Figure 6:**
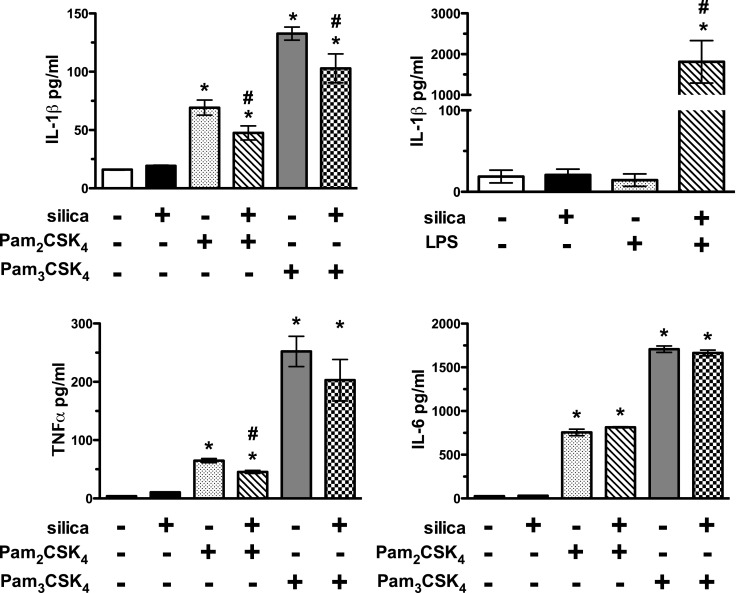
**Silica reduced IL-1β, but not IL-6, levels in response to synthetic diacylated and triacylated lipoproteins *in vitro***. Macrophages derived from the bone marrow of wild-type C57Bl/6 mice were exposed to media or silica (100 μg/ml) for 24 h and subsequently treated with fluorescently labeled Pam_2_CSK_4_ and Pam_3_CSK4 for 2 h. Cell-free supernatants were analyzed for the presence of the inflammatory cytokines IL-1β and IL-6. As expected, activation of naïve macrophages with Pam_2_CSK_4_ and Pam_3_CSK4 upregulated IL-1β and IL-6 levels. SiO_2_ alone induced a slight, but not significant, increase in IL-1β. Although SiO_2_ reduced the levels of IL-1β, it had no effect on the levels of IL-6 induced by TLR activation. Results are means ± SEM (*n* = 4). *p < 0.05 compared to media.

Given that the observed reduction in TLR expression correlated with a decrease in lipoprotein uptake, we next determined if this resulted in functional changes by analyzing the inflammatory response of BMM to SiO_2_ plus or minus synthetic triacylated and diacylated lipoproteins. We chose to focus on the trifecta of innate immune cytokines because of both lipoproteins are anticipated to induce the maturation and release of IL-1β, and to trigger the release of TNFα and IL-6 (likely *via* the activation of NF-kb signaling pathways). As anticipated, bone marrow-derived macrophages recognized and responded to synthetic triacylated and diacylated lipoproteins by increasing levels of IL-1β, TNFα, and IL-6 found in the tissue culture supernatant relative to media alone, whereas exposure to SiO_2_ alone resulted in little to no change (Figure 6). Although SiO_2_ exposure reduced the levels of IL-1β and TNFα present in the tissue culture supernatant in response to stimulation with either Pam_2_CSK_4_ or Pam_3_CSK4, it had no effect on the secretion of IL-6 (Figure 6). Furthermore, as a positive control, exposure to SiO_2_ plus 10 ng/ml LPS appears to have activated the Nlrp3 inflammasome, thus resulting in enhanced IL-1β secretion relative to either stimulus alone (Figure 6) and supporting the finding that TLR4 expression remains unchanged by SiO_2_ exposure. Finally, although we analyzed the tissue culture supernatants for the presence of IL-10, the levels detected were at or below the limit of detection of the assay.

## Discussion

Silicosis, the most prevalent of the pneumoconioses, is caused by inhalation of crystalline SiO_2_ particles. In addition to its importance as an occupational disease, silicosis or even exposure to SiO_2_ without established disease is associated with increased risk of developing many pulmonary and systemic comorbidities: chronic obstructive pulmonary disease, lung cancer, tuberculosis, non-tuberculous mycobacteria-related diseases, glomerulonephritis, rheumatoid arthritis, scleroderma, and other systemic autoimmune diseases. Although the epidemiological link between silicosis and tuberculosis has been acknowledged for decades, the cellular and molecular mechanisms underlying this increased risk remain largely unknown. As the first line of defense in the alveolar spaces, AM recognize and respond to inhaled pathogens and particulates, likely through interactions with PRRs, resulting in activation of NLRP3 inflammasome among many other signaling pathways. The primary objectives of this investigation were to (1) uncover if SiO_2_ modifies the profile of select PRRs expressed on macrophages and (2) examine the interactions between SiO_2_-exposed macrophages and Pam_3_CSK_4_ and Pam_2_CSK_4_: synthetic diacylated and triacylated lipopeptide ligands, which mimic bacterial cell wall components recognized by TLR2/6 and TLR2/1, respectively. The results from this study suggest that SiO_2_ interferes with the ability of macrophages to appropriately respond to bacterial ligands by downregulating the expression of TLR2 in a CD204-dependent, but inflammasome-independent manner.

The lung is constantly exposed to potentially harmful pathogens, including airborne particulates and microorganisms. Numerous studies have established that macrophages (alveolar and interstitial) are key orchestrators of pulmonary immunity and the prototypical host for diverse pathogens – including SiO_2_ and bacterial pathogens. In the steady state, the ability of macrophages to generate an inflammatory response is tightly regulated to ensure that lung injury does not occur, thus preserving alveolar physiology and gas exchange. By contrast, in response to insult, macrophages are responsible for the uptake and clearance of a wide variety of environmental contaminants (e.g., crystalline silica), as well as phagocytizing and eliminating bacteria (e.g., Mtb). Macrophage responses to airborne particulates and microorganisms ranges from ingestion and clearance with minimal inflammation to massive secretion of inflammatory mediators (e.g., cytokines and reactive oxygen species) and recruitment and/or activation of other innate and adaptive immune cell types. Although freshly isolated AMs most closely represent the *natural* state, bone marrow-derived macrophages are a widely used and accepted model system because of the relative simplicity of the isolation procedure, the high numbers of cell yielded, and the consistency of the cellular response to immune activation. In this study, both freshly isolated alveolar and bone marrow-derived macrophages were utilized to verify findings based on assay specific needs.

Alveolar macrophages are the first line of defense in the alveolar spaces against inhaled pathogens, and also serve to limit inflammation and minimize injury to preserve lung function. Because PRRs, such as C-type lectin receptors, SRs, TLRs, NOD-like receptors, and RIG-I like receptors, play a prominent role in the activation of AMs and subsequent cross-talk with innate and adaptive immune cells, how a macrophage reacts to a given stimulus depends greatly on the diverse range of PRRs expressed on the cell’s surface (43, 44). In the case of concomitant or sequential exposure to two distinct pathogens, the capacity of macrophages to recognize, phagocytose, and appropriately respond to a second stimuli may be compromised by stimulant-induced changes in the profile of PRRs (45) – thus altering susceptibility to disease. TLRs, which recognize microbial molecules, are major triggers of innate responses (e.g., enhanced costimulatory molecule expression, cytokine secretion, production of reactive oxygen species, and antimicrobial mediators) and thus modulate adaptive immunity by influencing macrophage functions. TLRs are important for host responses to Mtb. In particular, TLR-2 activation has been shown to play a prominent role in eliciting appropriate immune responses to *Mycobacterium avium* or Mtb, as well as to bacterial products, such as lipoarabinomannan, lipoprotein, and phosphatidylinositol mannosides (28, 46–51). Furthermore, associations between TLR2 gene polymorphisms and tuberculosis have been reported for a range of different human populations (52, 53), suggesting that changes in TLR2 expression may be involved in susceptibility to disease. In this study, we tested the hypothesis that exposure to SiO_2_ triggers phenotypic changes in AMs – recognized as differences in the PRR profile. Following acute SiO_2_ exposure (≤4 h) in C57BL/6 mice, we ascertained that live F4-80^+^CD11c^+^ AMs downregulate the expression of TLR2 using multi-color flow cytometry. These results were confirmed using F4-80^+^CD11b^+^ murine bone marrow-derived macrophages, and more importantly shown to be dependent on the presence of the class A SR, CD204, but not the NLRP3 inflammasome. These findings support the importance of interactions between SiO_2_ and CD204 (40) and further link the SiO_2_–CD204 interface in macrophages to the inflammatory response to ligands acting at TLR2. Of note, Chávez-Galán et al. recently reported that human monocyte derived macrophages and a human macrophage cell line (THP-1) also respond to SiO_2_ by downregulating the expression of TLR2 in a dose-dependent manner (54). Although this study did not explore the role of SRs in this process, their results suggest that SiO_2_ may impair the ability of human macrophages to control intracellular bacterial growth (54). These results led us to assess whether SiO_2_ altered the expression of other PRRs (e.g., TLR4, TLR5, TLR6, and DC-SIGN) involved in innate immunity to various pathogens. Although acute exposure to SiO_2_ decreased TLR6 expression on AMs, we have not yet examined whether this reduction in protein expression is dependent on CD204. These data suggest that interaction between SiO_2_ and CD204 may regulate the responsiveness of antigen-presenting cells to TLR2 activation. Previous studies have also observed connections between CD204 and TLR4 signaling (55, 56); although little is known about the biochemical nature of such interactions.

Recognition of TLR ligands results in immune activation, which can be measured as enhanced costimulatory molecule expression, cytokine secretion, production of reactive oxygen species, and antimicrobial mediators. Previous studies from our laboratory group demonstrated that SiO_2_ downregulates the expression of costimulatory molecules on murine bone marrow-derived dendritic cells. These results lead us to examine whether SiO_2_-induced changes in TLR2 expression may result in aberrant response to bacterial ligands *in vitro*. Furthermore, SiO_2_ exposure reduced uptake of fluorescently labeled synthetic diacylated and triacylated lipoproteins recognized by TLR2/6 and TLR2/1 in both C57Bl/6 wild-type and CD204^−/−^ derived macrophages. Several possibilities arise from this incongruity that CD204^−/−^ macrophages do not downregulate TLR2 expression, yet exhibit reduced ability to uptake rhodamine-labeled Pam_2_CSK_4_ and Pam_3_CSK_4_. CD204^−/−^ macrophages may have enhanced expression of other PRRs (e.g., biological compensation) (57), the reduced levels of TLR2 may not be responsible for changes in diacylated and triacylated ligand uptake, and SiO_2_ may alter signaling molecules down stream of the receptor, resulting in the same net effect. Interestingly, we also observed attenuated levels of the inflammatory cytokines IL-1β and TNFα, but not IL-6, in the culture supernatants. These results are intriguing and suggest that SiO_2_ disrupts more downstream signal transduction events pertinent to the maturation and secretion of cytokines.

In summary, uptake of SiO_2_ downregulates the expression of select PRRs on AMs, as well as their ability to recognize, uptake, and respond to specific ligands. We hypothesize that these changes in AM phenotype may play a role their ability to appropriately respond to a secondary pathogen such as mycobacteria following SiO_2_ exposure. Our data suggest that SiO_2_, interacting with CD204, does not indiscriminately alter expression of all PRRs, but rather may amend signaling components involved in macrophage activation. Moreover, our data identify CD204 as an important partner for TLR2 on macrophages for the production of inflammatory mediators in response to bacterial stimuli. Future experiments may shed light on the relationship between reduced TLR2 expression and immunity to MTB infection later on.

## Author Contributions

CB designed the study, coordinated the experiments, prepared the figures, and composed the manuscript. BS performed the tissue culture, flow cytometry, and ELISA experiments under the direction of CB. FJ contributed data relative to Figures 4 and 5. GB and DS contributed to the manuscript preparation and critical revision. All authors have read and approved the final version of the manuscript.

## Conflict of Interest Statement

The authors declare that the research was conducted in the absence of any commercial or financial relationships that could be construed as a potential conflict of interest.
